# Incidence, characteristics and prognosis of acute kidney injury in Cameroon: a prospective study at the Douala General Hospital

**DOI:** 10.1080/0886022X.2017.1419970

**Published:** 2017-12-29

**Authors:** Marie Patrice E. Halle, Ndjifoum Moselle Chipekam, Gérard Beyiha, Hermine Fouda, Aminata Coulibaly, Romuald Hentchoya, Folefack Francois Kaze, Namme Henry Luma, Gloria Ashuntantang

**Affiliations:** a Faculty of Medicine and Pharmaceutical Sciences, University of Douala, Douala, Cameroon;; b Department of Internal Medicine, Douala General Hospital, Douala, Cameroon;; c Intensive Care Unit, Douala General Hospital, Douala, Cameroon;; d Faculty of Medicine and Biomedical Sciences, University of Yaounde, Yaounde, Cameroon

**Keywords:** Acute kidney injury, incidence, prognosis, Douala, Cameroon

## Abstract

**Objective:** There are limited data on AKI in sub-Saharan Africa. We aim to determine the incidence, characteristics and prognosis of AKI in Cameroon.

**Patients and methods:** A prospective study including all consenting acute admissions in the internal medicine and the ICU of a tertiary referral hospital in Cameroon from January 2015 to June 2016. Serum creatinine assay was done on admission, days 2 and 7 to diagnose AKI. For patients with AKI, serum creatinine was done on discharge, days 30, 60 and 90. AKI was defined according to the modified KDIGO 2012 criteria as an increase or decrease in serum creatinine of 3 mg/l or greater, or an increase of 50% or more from the reference value obtained at admission or the known baseline value. AKI severity was graded using KDIGO2012 criteria. Outcome measures were renal recovery, mortality and causes of death. Renal recovery was complete if serum creatinine between the first 90 days was less than baseline or reference, partial if less than diagnosis but not baseline or reference, no-recovery if creatinine did not decrease or if the patient remained on dialysis.

**Results:** Of the 2402 patients included, 536 developed AKI giving a global incidence of 22.3% and annual incidence of 15 per 100 patients-years. Of the 536 patients with AKI, 43.3% were at stage 3, 54.7% were males, median age was 56 years. Pre-renal AKI (61.4%) and acute tubular necrosis (28.9%) were the most frequent forms. Main etiologies were sepsis (50.4%) and volume depletion (31.6%). Renal outcome was unknown in 34% of patients. Of the 354 patients with known renal function at 3 months, 84.2% recovered completely, 14.7% partially and 1.1% progressed to CKD. Global mortality rate was 36.9% mainly due to sepsis.

**Conclusions:** AKI is frequent in our setting, mainly due to sepsis and hypovolemia. It carries a poor prognosis.

## Introduction

Acute kidney injury (AKI) a common worldwide problem is defined as a rapid decline or loss of kidney function. It is encountered in multiple settings but remains a poorly diagnosed disease in the world [1]. The global burden of AKI is estimated at 13.3 million cases per year, with 85% from low-middle-income countries (LMIC) [1,2]. Limited data on the incidence are available worldwide and the data vary widely across studies depending on the setting and the populations investigated. In a recent meta-analysis on world incidence of AKI, including 154 studies with only two from Africa, the reported incidence was 21% in adults and 33.7% in children [3].

In developed countries, hospital acquired AKI is the most frequent form with an incidence of 7–18% and preponderance for elderly patients [4–8]. In contrast, in low income countries (LIC) including Sub-Saharan Africa (SSA), AKI commonly occurs in the community, affecting mostly young adults and children [9–11]. The etiology of AKI in LMIC depend on geographical location and many are preventable. A recent meta-analysis by Wasiu et al. reported that the common causes of AKI in adults in SSA were infections (28%), nephrotoxins (18%), pregnancy related (16%), glomerular disease (8%) and hypovolemia (5%) [11]. The majority of AKI cases are therefore preventable or can be managed by simple measures. Despite advances in medical technique, AKI remains under diagnosed especially in SSA. When diagnosed late, AKI has adverse effects for the individual in general. It is associated with increased length of hospital stay and high cost [12]. The duration and severity of AKI is a risk factor for the development of complications such as a 10-fold increase risk of chronic kidney disease (CKD) and a 3-fold risk of end stage kidney disease (ESKD) [13–15]. Also, AKI is associated with a high risk of death with an annual mortality rate estimated to be greater than prostate cancer, breast cancer, heart failure and diabetes [1].

In SSA, the outcome of patients with AKI is very poor with an overall mortality of 32% in adults. This is extremely high compared to the pooled world mortality of 23.9%. This mortality increases with the severity of AKI which is estimated at 50–60% amongst patients requiring renal replacement therapy (RRT) and to 82% in those in need for dialysis who could not receive it [3,11]. This high mortality in SSA is due to the late presentation of patients with severe disease in the hospital, the non-availability of RRT and the inability to afford treatment as health care costs are covered by out of pocket payment in most SSA countries [10,11,16–20]. Data on the epidemiology of AKI in SSA in general and in Cameroon in particular are limited, but the prevalence of AKI is estimated to be higher than that in developed countries [3,21]. The incidence in this setting is difficult to know due to lack of national registries. Because AKI is associated with high mortality and treatment costly, identifying patients early and intervening to avoid RRT is necessary. Therefore, the objectives of the present study were to determine the incidence, characteristic and outcomes of AKI in Cameroon.

## Patients and methods

### Study setting

This study was conducted in the intensive care unit (ICU) and internal medicine (IM) ward of the Douala General hospital (DGH). This is the main tertiary reference hospital of the country and a teaching hospital with 320 beds located in the littoral region of Cameroon with approximately three million inhabitants. The hospital has the only public hemodialysis center of the region and therefore the referral hospital for patients with kidney disease. It has a central laboratory for all inpatient and outpatient samples.

### Study design

This was an observational prospective study including all patients admitted in the two units from January 2015 to June 2016 (18 months). For each consenting patient, serum creatinine assay was done on admission, days 2 and 7 to diagnose AKI. For patients with AKI, serum creatinine assay was repeated on discharge, days 30, 60 and 90. Creatinine was measured using the Jaffe kinetic method with a spectrophotometer (BIOMERIEUX^®^, FRANCE) throughout the study period.

Other variables collected were: socio demographic information such as age and gender; clinical data including co morbidities, primary diagnosis, signs and symptoms, of AKI, length of hospital stay and outcomes. Outcomes measures were the need of dialysis, renal recovery and patient mortality.

### Definition of operational terms

AKI was defined according to the modified KDIGO 2012 criteria [22] as an increase or decrease in serum creatinine of 0.3 mg/dl or greater, or an increase of 50% or more from the reference value obtained at admission or the known baseline value. AKI severity was graded using KDIGO 2012 criteria [23]. Sepsis was defined as the presence a systemic inflammatory response (fever >38 °C, high white cell count at presentation) an increased C-reactive protein level due to suspected or proven infection (by positive culture or tissue stain) caused by any pathogen or a clinical syndrome associated with a high probability of infection [24]. AKI was community-acquired if patients first presented to the hospital with AKI. Renal recovery was defined as complete if serum creatinine between the first 90 days was equal to or lower than baseline or reference value. Renal recovery was partial if serum creatinine was lower than diagnosis value but not to baseline or reference, and no-recovery if serum creatinine did not decrease or if the patient remained on dialysis.

The diagnosis of acute tubular necrosis was done based on medical history, presence of risk factors, urine indices when available and recovery with a polyuric phase. While for pre-renal AKI medical history, the presence of risk factors, urea/creatinine ratio >20 in the absence of confounders and urine indices when available were used. Obstructive AKI was diagnosed based on history of acute oliguria or anuria and presence of dilatation of urinary tract on ultrasound.

Nephrotoxic AKI was diagnosed based on a history of ingestion of known nephrotoxic drug(s) (NSAID, Angiotensin converting system inhibitors, Cisplatine, Aminoside, Iodine contrast) or an herbal concoction.

Hypertension was considered in any patients on blood pressure lowering medication or blood pressure greater than 140/90 mmHg, while hypotension was blood pressure less than 90/60 mmHg.

Ethical approval was obtained from the ethical committee board of the Douala University and administrative authorization from the DGH.

### Statistical analysis

Data were analyzed using Stat view version 5.0 for windows (SAS Institute, Inc., IL, USA). Continuous variables were presented as mean ± standard deviation and/or as median (inter-quartile range). Categorical variable were expressed as percentages. Chi-squared test was used to compare categorical data and *t*-test or Mann–Whitney test to compare continuous data. A *p* values <.05 was considered significant.

## Results

**Figure 1.**
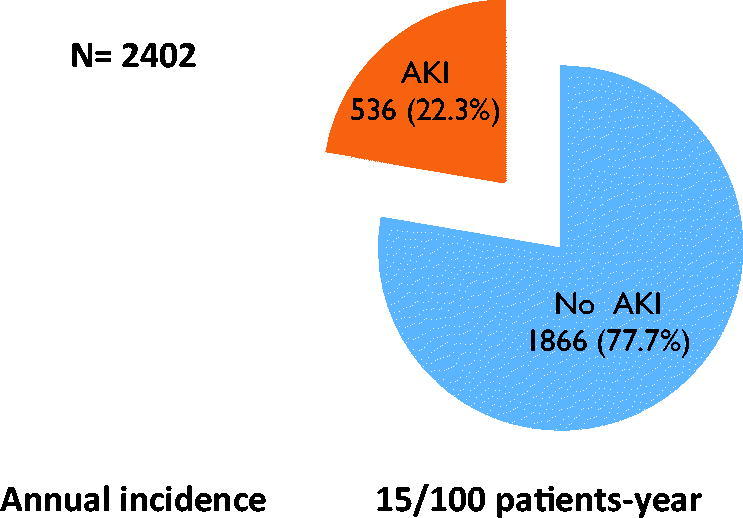
Incidence of AKI.

From January 2015 to June 2016, a total of 2402 patients were admitted with 580 from ICU and 1822 in IM of whom AKI occurred in 536. This corresponded to a global incidence of 22.3% and an annual incidence of 15 per 100 patients-years (Figure 1). Of the 536 patients with AKI, 54.7% were males and the median age was 56 years (14–95). Median length of stay was 8 days (1–54). Main comorbidities were: hypertension (32.2%), diabetes (17.6%), HIV infection (12.6%) and cancer (9.3%). A total of 6.1% (39/536) patients had a known history of CKD (Tables 1 and 2).

**Table 1.** General characteristics of AKI patients.

Variables	Total (%) *N***** ****=**** ****536	Male (%) *N***** ****=**** ****293	Female (%) *N***** ****=**** ****243	*p*
Age (years)				
Mean ± SD	54.71 ± 18.41	55.53 ± 17.39	53.72 ± 19.56	.256
Unit of hospitalization				
Internal medicine	407 (75.90)	230 (42.90)	177 (33.00)	.170
Intensive care unit	129 (24.10)	63 (11.80)	66 (12.30)	
Length of hospital stay (IQ)				
Median (IQ)	8 (1–41)	8 (1–39)	8 (1–41)	.909
Comorbidity				
Hypertension	205 (32.20)	123 (19.30)	82 (12.90)	.059
Diabetes mellitus	112 (17.60)	64 (10.00)	48 (7.60)	.584
HIV	80 (12.60)	38 (5.90)	42 (6.70)	.152
Cancer	59 (9.30)	24 (3.80)	35 (5.50)	1.000
Hepatitis B/C	51 (8.00)	38 (5.90)	13 (2.10)	**.003**
Heart failure	47 (7.40)	26 (4.10)	21 (3.30)	.946
Known CKD	39 (6.10)	26 (4.10)	13 (2.00)	.096
Liver cirrhosis	22 (3.40)	16 (2.50)	6 (0.80)	.085
Gout	20 (3.10)	15 (2.30)	4 (0.80)	1.000
Systemic lupus	2 (0.30)	0 (0.00)	2 (0.30)	1.000

HIV: human immunodeficiency virus; CKD: chronic kidney disease.

Bold indicates the significant *p* values.

**Table 2.** Clinical and biological characteristics of the study population.

Variables	Total (%)	Male (%)	Female (%)	*p*
Urine dipstick on admission (n = 350)				
Proteinuria	242 (43.50)	124 (22.30)	118 (21.20)	.067
Hematuria/hemoglobinuria	138 (24.80)	63 (11.30)	75 (13.50)	**.003**
Leucocyturia	140 (25.20)	51 (9.20)	89 (16.0)	**<.001**
Nitrites	36 (6.50)	12 (2.20)	24 (4.30)	**.006**
Specific gravity (n = 350)				
Between 1010 and1020	85 (24.30)	41 (11.70)	44 (12.60)	
>1020	167 (47.70)	108 (30.80)	59 (16.90)	**.023**
<1010	98 (28.00)	45 (12.90)	53 (15.10)	
Urine output on admission (n = 437)				
<100 ml/day	183 (41.80)	99 (22.60)	84 (19.20)	**.047**
100–500 ml/day	187 (42.80)	88 (20.10)	99 (22.70)	
500–2500 ml/day	58 (13.30)	33 (7.60)	25 (5.70)	
>2500 ml/day	9 (2.10)	7 (1.60)	2 (0.50)	
Blood pressure (mmHg)				
Hypotension	293 (54.70)	169 (31.50)	124 (23.20)	.350
Normal	184 (34. 30)	94 (17.50)	90 (16.80)	
Hypertension	59 (11.00)	30 (5.60)	29 (5.40)	
Biology (mean ± SD)				
Urea (*n* = 536)	1.29 ± 0.89	1.29 ± 0.97	1.28 ± 0.78	.976
Creatinine (*n* = 536)	48.44 ± 52.76	47.25 ± 53.94	49.89 ± 51.38	.565
Hemoglobin (*n* = 531)	9.49 ± 2.98	9.77 ± 3.17	9.15 ± 2.69	**.016**
White blood count (*n* = 531)	12.98 ± 21.15	12.97 ± 23.48	12.99 ± 17.99	.988
CRP (*n* = 449)	147.77 ± 112.68	146.03 ± 104.91	149.88 ± 121.65	.727
Sodium (*n* = 499)	136.78 ± 9.27	136.72 ± 8.23	136.86 ± 9.80	.626
Potassium (*n* = 499)	4.53 ± 1.25	4.52 ± 1.19	4.54 ± 1.31	.861
Bicarbonate (*n* = 83)	18.57 ± 6.72	18.93 ± 7.41	18.18 ± 7.41	.614

CRP: C-reactive protein.

Bold indicates the significant *p* values.

AKI was community-acquired in 93.3% (500/536) and 43.3% (232/536) had AKI stage 3 (Table 3). Pre-renal AKI with a frequency of 61.4% (329/536) and acute tubular necrosis 28.9% (155/536) were the most frequent forms. AKI was due to obstruction in 4.10% (22/536) of patients. Main etiologies of AKI were sepsis/bacterial infection, 310/536 (50.4%) and volume depletion 195/536 (31.6%). Nephrotoxins accounted for 62/536 (10.1%), pelvic tumor for 3.0% (18/536) and gynecologic/obstetric causes for 7.1% (38/536). The main sources of infection were urinary 25.1% (88/310), digestive 21.5% (75/310) and pulmonary 18.3% (64/310) (Table 4). Conversely, volume depletion was mainly due to hemorrhage 23.7% (48/186), gastroenteritis 23.1% (47/186), decompensated diabetes 21.7% (44/186) and heart failure 17.7% (36/186) (Table 4). Main causes of obstetric AKI were cervical cancer (34.2%), eclampsia (28.8%) and post-partum hemorrhage (21.1%) while herbal remedies (32.3%) were the main toxins (Table 5).

**Table 3.** Nature, mechanism, severity and etiology of AKI amongst participants.

Variables	Total (%)	Male (%)	Female (%)	*p*
Nature				
Community acquired	500 (93.30)	278 (51.90)	222 (41.40)	.099
Hospital acquired	36 (6.70)	15 (2.80)	21 (3.90)	
Mechanism				
Pre renal	329 (61.40)	195 (36.40)	134 (25.00)	**.004**
Acute tubular necrosis	155 (28.90)	84 (15.70)	71 (13.20)	
Glomerular	6 (1.10)	2 (0.40)	4 (0.70)	
Interstitial	14 (2.60)	3 (0.60)	11 (2.00)	
Vascular	10 (1.90)	3 (0.60)	7 (1.30)	
Obstructive	22 (4.10)	6 (1.10)	16 (3.00)	
Severity AKI				
1	176 (32.80)	114 (21.30)	62 (11.50)	**.005**
2	128 (23.90)	65 (12.10)	63 (11.80)	
3	232 (43.30)	114 (21.30)	118 (22.00)	
Etiologies				
Sepsis/bacterial infection	310 (50.40)	173 (28.10)	137 (22.30)	
Volume depletion	195 (31.60)	109 (17.70)	86 (13.90)	
Nephrotoxins	62 (10.10)	40 (6.50)	22 (6.40)	
Obstruction	22 (3.60)	5 (0.80)	17 (2.80)	
Eclampsia/HELLP	11 (1.80)	0 (0.00)	11 (1.80)	
Multiple Myeloma	5 (0.80)	3 (0.50)	2 (0.30)	
Malignant hypertension	5 (0.80)	3 (0.30)	2 (0.20)	
Systemic lupus	3 (0.50)	0 (0.00)	3 (0.50)	
Lymphoma	1 (0.20)	1 (0.20)	0 (0.00)	
Unknown	1 (0.20)	1 (0.20)	0 (0.00)	

Bold indicates the significant *p* values.

**Table 4.** Causes of sepsis/bacterial infection, volume depletion and obstruction amongst the study population.

Variables	Total (%)	Male (%)	Female (%)	*p*
Sepsis/bacterial infection	350	190 (54.30)	160 (45.70)	
Urinary	88 (25.10)	40 (11.40)	48 (13.70)	
Digestive	75 (21.50)	47 (13.40)	28 (8.10)	
Pulmonary	64 (18.30)	38 (10.90)	26 (7.40)	.403
Unknown origin	49 (14.00)	30 (8.60)	19 (5.40)	
Cutaneous	37 (10.60)	19 (5.40)	18 (5.20)	
Meningitis	18 (5.10)	5 (1.40)	13 (3.70)	
Septicemia	13 (3.70)	9 (2.60)	4 (1.10)	
Gynecology	4 (1.10)	0 (0.00)	4 (1.10)	
Endocarditis	1 (0.30)	1 (0.30)	0 (0.00)	
Chronic otitis	1 (0.30)	1 (0.30)	0 (0.00)	
Volume depletion	203	112 (55.20)	91 (44.80)	
Hemorrhage	48 (23.70)	27 (13.30)	21 (10.40)	
Gastroenteritis	47 (23.10)	26 (12.70)	21 (10.40)	
Diabetes mellitus	44 (21.70)	21 (10.40)	23 (11.30)	.701
Heart failure	36 (17.70)	19 (9.40)	17 (8.30)	
Liver cirrhosis	14 (6.80)	9 (4.40)	5 (2.40)	
Diuretics	6 (2.90)	5 (2.40)	1 (0.50)	
Burn	4 (2.00)	3 (1.50)	1 (0.50)	
Intestinal occlusion	2 (1.00)	1 (0.50)	1 (0.50)	
NSAID	1 (0.50)	1 (0.50)	0 (0.00)	
Unspecified	1 (0.50)	0 (0.50)	1 (0.00)	
Obstructive AKI	22	5 (22.70)	17 (77.30)	
Pelvic tumor	18 (81.80)	5 (22.70)	13 (59.10)	
Cervical cancer	13 (2.10)	0 (0.00)	13 (2.10)	
Prostate cancer	4 (0.70)	4 (0.70)	0 (0.00)	
BPH	1 (0.20)	1 (0.20)	0 (0.00)	**.004**
Ureters ligation	2 (9.20)	0 (0.00)	2 (9.20)	
Retroperitoneal ibrosis	1 (4.50)	0 (0.00)	1 (4.50)	
Renal stones	1 (4.50)	0 (0.00)	1 (4.50)	

NSAID: non-steroidal anti-inflammatory drugs; BPH: benign prostatic hyperplasia.

Bold indicates the significant *p* values.

**Table 5.** Nephrotoxins and obstetrical/gynecological causes of AKI amongst study population.

Variables	Total (%)	Male (%)	Female (%)	*p*
Nephrotoxins	62 (100%)	40 (64.50)	22 (35.50)	.836
Herbal remedies	20 (32.30)	13 (21.00)	7 (11.30)	
Unspecified drugs	11 (17.80)	7 (11.30)	4 (6.50)	
Rhabdomyolysis	9 (14.50)	7 (11.30)	2 (3.20)	
Black water fever	8 (12.90)	6 (9.70)	2 (3.20)	
Tumor lysis syndrome	5 (8.10)	3 (4.90)	2 (3.20)	
Hemolysis, others	3 (4.80)	1 (1.60)	2 (3.20)	
Iodine contrast product	3 (4.80)	2 (3.20)	1 (1.60)	
Amphotericine B	2 (3.20)	0 (0.00)	2 (3.20)	
ACE inhibitors	1 (1.60)	1 (1.60)	0 (0.00)	
Obstetric or gynecology causes	38 (100)	0 (0.00)	38 (100.00)	
Cervical cancer	13 (34.20)	0 (0.00)	13 (34.20)	
Eclampsia/HELLP	11 (28.80)	0 (0.00)	11 (28.80)	
Post-partum hemorrhage	8 (21.10)	0 (0.00)	8 (21.10)	
Septic abortion	2 (5.30)	0 (0.00)	2 (5.30)	
Endometritis	2 (5.30)	0 (0.00)	2 (5.30)	
Bilateral ureters ligation post caesarian section	2 (5.30)	0 (0.00)	2 (5.30)	

ACE: angiotensin converting enzyme.

Amongst the 54 (10.1%) patients with indication for dialysis, 15 (2.8%) could not receive the treatment due to lack of appropriate equipment and denial (Table 6). Renal recovery could not be determined in 34% (182/536) either because the patient died in the acute phase (137/182) or was lost to follow-up (45/182). Of the 354 patients with known renal function at 3 months, 84.2% (298/354) recovered completely, 14.7% (52/354) partially and 1.1% (4/354) progressed to CKD (Table 6). At 3 months a total of 198 patients died giving a global mortality rate of 36.9%. Sepsis was the leading cause of death in 56.9% (119/209) (Table 7).

**Table 6.** Management and outcomes of AKI patients.

Variables	Total (%)	Male (%)	Female (%)	*p*
Use of vasoactive drugs	71 (13.20)	39 (7.30)	32 (5.90)	.988
Blood transfusion	145 (27.10)	72 (13.50)	73 (13.60)	.202
Dialysis indication	54 (10.10)	26 (4.90)	28 (5.20)	
Dialysis done	39 (7.30)	15 (2.30)	24 (5.00)	.296
Reasons for non-realization of dialysis (n = 15)				
Inadequate equipment	10 (66.80)	7 (46.80)	3 (20.00)	1.000
Denial	3 (20.00)	3 (20.00)	0 (10.00)	1.000
Early death	2 (13.20)	1 (6.60)	1 (6.60)	1.000
Renal recovery at 3 months known (*n* = 354)				
Complete recovery	298 (84.20)	158 (44.60)	140 (39.60)	.613
Partial recovery	52 (14.70)	30 (8.50)	22 (6.20)	
No recovery	4 (1.10)	4 (1.10)	0 (0.00)	
Unknown (*n* = 182)				
Early death	137 (75.30)	78 (42.80)	59 (32.50)	.613
Loss to follow-up	45 (24.70)	23 (12.60)	22 (12.10)	
Patients outcomes				
Survival	269 (50.20)	144 (26.90)	125 (23.30)	.502
Death	198 (36.90)	112 (20.90)	86 (16.00)	
Loss to follow-up	69 (12.90)	37 (6.90)	32 (6.00)	

**Table 7.** Presumed causes of death in the study population.

Variables	Total *n* = 209		Male *n* = 112	Female *n* = 86	*p*
Presumed causes of death					
Sepsis		119 (56.90)	67 (32.00)	52 (24.90)	.328
Multi organ dysfunction		22 (10.60)	11 (5.30)	11 (5.30)	
Hemorrhage		19 (9.10)	11 (5.30)	8 (3.80)	
Cancer		18 (8.60)	9 (4.30)	9 (4.30)	
Liver cirrhosis		9 (4.30)	8 (3.80)	1 (0.50)	
Pulmonary embolism		8 (3.80)	4 (1.90)	4 (1.90)	
Cardiogenic shock		8 (3.80)	3 (1.40)	5 (2.40)	
Absence of dialysis		5 (2.40)	3 (1.40)	2 (1.00)	
Eclampsia		1 (0.50)	0 (0.00)	1 (0.50)	

## Discussion

To the best of our knowledge, no study on AKI has been performed prospectively in our country. This is the first study to determine the incidence of AKI in Cameroon. The results showed that AKI is frequent with a global incidence of 22.3% and annual incidence of 15/100 patient-year. AKI affects young adults, is mostly community acquired (93.3%) and stage 3 accounts for almost half of case. Pre-renal AKI and acute tubular necrosis from sepsis were the most frequent forms. Dialysis was indicated in 54 patients, but was not done in 15 patients due to lack of adequate materials. Renal outcome was unknown in 1/3 of participants. In those with known renal function, recovery was complete in more than 4/5 and more than 1/3 patients died.

### Incidence of AKI

AKI is a public health problem worldwide and in SSA in particular. It is a potentially preventable and reversible disease, often asymptomatic and is a common condition amongst hospitalized patients. In the present study, the global incidence of AKI was 22.3% and an annual incidence of 15/100 patients-years.

Worldwide incidence varies widely across studies depending on the setting and the populations investigated. Data on the incidence of AKI in SSA are very scanty due to lack of registries. Our result was higher compared to the recent reported pooled world incidence of 21% in adults [3] and also much higher as the results of recent hospital based studies in the developed world that reported AKI in 3.2–9.6% of admissions [25,26]. In our study, 93.3% of AKI cases were community acquired. This is in accordance with reported findings in LIC where contrary to developed countries, AKI is mostly community acquired and affects more young adults and children [9,11,27]. One explanation of this figure may be the underlying causes of AKI in LIC such as sepsis, hypovolemia due to diarrhea and nephrotoxic drugs. All these factors are prevalent in the community [11,17,28]. Almost half of our patients were at AKI stage 3 with various symptoms; this is mainly due to the severity of the underlying disease and late presentation of patients in our hospital that is a reference hospital for severe medical cases in the region. Our result is in accordance with reports in SSA [11].

### Etiology of AKI

The etiology of AKI in LMIC is dependent on geographical location. A recent meta-analysis from Wasiu et al. reported that the two leading causes in SSA were infections and nephrotoxin [11]. In the present study, pre-renal AKI (61.4%) and acute tubular necrosis (28.9%) were the most frequent forms. Sepsis from bacterial infection was the main precipitating factor followed by hypovolemia mainly from dehydration and hemorrhage. Similar findings were reported in developed and developing countries [18,29–33]. The main source of infection in our study population was the urinary tract (88/310), followed by gastroenteritis (75/310) and pneumonia (64/310). These three sources have been reported in the literature but with differences in proportion [34–36]. Pneumonia was the leading cause in the study of Madav et al. in Nepal followed by gastroenteritis and urinary tract infection [35]. In contrast, gastroenteritis was the first etiology in the studies of Praskash et al. (60%) in India and Arogundade et al. (36.9%) in Nigeria [17,28]. Toxic AKI is common in SSA with a global prevalence of 18% [11]. We found that 62 (10.1%) cases of AKI were due to nephrotoxins mainly herbal remedies (20 patients). This is similar to reported findings in LIC [27,37,38]. We found 22 cases (4.10%) of obstructive AKI, similar to the reported prevalence in SSA (5%), of Singhal et al. (5%) and Nagamani et al. (4%) [11,32,39]. In SSA, the major causes of obstruction are renal stone, prostate hypertrophy and less malignancy [11]. In our study, malignancy mainly solid pelvic cancer (18 cases) was the leading cause of obstruction. This high proportion of cancer can be explained by the fact that our hospital has a referral center for oncology included in the internal medicine ward and patients are usually referred at late stage of the disease in that setting with complications [40]. The estimated prevalence of obstetric AKI in SSA is 16% mainly due to septic abortion, eclampsia and post-partum hemorrhage [11]. With the improved maternal care, the incidence has decreased. We found an incidence of obstetric AKI of 7.1% (38 patients) with eclampsia (11 patients) post-partum hemorrhage (eight cases) and sepsis (four patients) being the major causes. This is similar to other studies in SSA [30,41].

### Dialysis and outcome

The pooled rate of dialysis requirement for AKI in the world was estimated at 2.3% [3]. In total, 10.1% (54/536) of our participants needed dialysis and this is much lower compared to most studies in SSA that reported 70% of adults in need of dialysis [11]. One major explanation of the low rate of dialysis need in the present study is that contrary to studies in SSA, this was a prospective study based on incidence. Patients were screened on admission and therefore AKI was diagnosed and managed earlier reducing the need of dialysis. Dialysis could not be realized in 15 patients (2.8%), patients mostly due to lack of adequate materials. Scarcity of resources is already known as a frequent reason for not providing dialysis in LIC [10,17,22,30,42]. For patients with known renal function at 3 months, full renal recovery was achieved in 298 (84.2%), partial in 52 (14.7%) and four (1.1%) progressed to CKD. Renal outcome is not routinely reported in studies from SSA and the true rate is unknown. Tariq et al. reported higher rate of full recovery (92.5%), partial (7%) and no recovery in 0.6% of patients [29]. Renal recovery was estimated at 55% in adult in the study of Wasiu et al. with 13% of patients with residual CKD [11]. In contrast our renal recovery rate was extremely high compared to one study in the same setting that reported full recovery only in 55.4% of patient [30]. This difference can be explained by the retrospective nature of that study and the small sample size (108 patients). Renal recovery could not be determined in 182 (34%) patients either because the patient died in the acute phase or was loss of follow-up similar to finding of Tariq et al. [29].

Despite advances in medical techniques, AKI is relatively common and still linked to adverse outcomes such as high in-hospital and long-term mortality rate [25,26,43] with a high risk for CKD development in those who survived [14]. The mortality rate in this study was estimated at 36.9% and this is extremely high compared to the pooled world AKI-associated all-cause mortality of 23.9% [3]. But our rate is comparable to the findings in SSA where the pooled rate in adults was estimated at 32% [11]. The high mortality rate could be explained mostly by the severity of the underlying disease and also the late referral and presentation of patients to the hospital with severe disease as HGD is the main tertiary referral hospital in Cameroon. The reasons for late presentation could be the silent evolution of AKI and especially the lack of funds as medical costs are covered by out of pocket payment for the majority in our setting and in most countries in SSA [16,17,20,42]. The high mortality rate associated with AKI is alarming. The International Society of Nephrology has set a goal ‘0 by 25’ that zero people should die of untreated AKI in the poorest parts of the world by 2025 (1, 2) and implementation of this program in low-resource settings will surely reduce the burden of AKI. In this study, sepsis from bacterial infection was responsible of more than half of death. This is in accordance with other findings in the literature where sepsis is reported as the major cause of death among patients with AKI especially in ICU [35,44]. Studies have reported significantly worse outcomes of patients with sepsis and AKI compared to non-septic AKI or sepsis alone [36,34,45–47].

### Strength and limitations

There are limited data addressing the epidemiology and causes of AKI in LIC. To our knowledge this is the first study that provides a convenience sample of the incidence of AKI in Cameroon. One limitation is that the study was carried out in a single referral tertiary hospital. Furthermore, urine indices were not recorded. But given the prospective nature of data collection and the population studied, we assume that these results are representative. It will bridge the gap of knowledge on AKI incidence and outcome in SSA, raise awareness of AKI and provide caregivers with knowledge to identify and adequately manage patients at risk. Consequently, preventable deaths associated with AKI could be reduced in our setting.

## Conclusions

Data on incidence of AKI in SSA are scanty and this study showed that AKI is a frequent condition in Cameroon; it is mostly community-acquired from bacterial infections and hypovolemia. Most patients recovered their renal function, but mortality rate was extremely high mainly due to sepsis. Prevention remains the key to reduce incidence and mortality due to AKI. This result may help health care providers for service planning and provide information to them for early action to prevent deterioration of renal function in hospitalized patients. The poor prognosis reported raises the need of implementing effective programs for AKI prevention, early detection and treatment in SSA and also determine factors contributing to this poor outcome as most etiologies of AKI in this setting are preventable.
